# ACKT: A Proposal for a Novel Score to Predict Prolonged Mechanical Ventilation after Surgical Treatment of Meningioma in Geriatric Patients

**DOI:** 10.3390/cancers13010098

**Published:** 2020-12-31

**Authors:** Elisa Scharnböck, Leonie Weinhold, Anna-Laura Potthoff, Niklas Schäfer, Muriel Heimann, Felix Lehmann, Erdem Güresir, Christian Bode, Andreas H. Jacobs, Hartmut Vatter, Ulrich Herrlinger, Matthias Schneider, Patrick Schuss

**Affiliations:** 1 Department of Neurosurgery, Center of Integrated Oncology (CIO) Bonn, University Hospital Bonn, 53127 Bonn, Germany; anna-laura.potthoff@ukbonn.de (A.-L.P.); muriel.heimann@ukbonn.de (M.H.); erdem.gueresir@ukbonn.de (E.G.); hartmut.vatter@ukbonn.de (H.V.); matthias.schneider@ukbonn.de (M.S.); patrick.schuss@ukbonn.de (P.S.); 2 Institute of Medical Biometry, Informatics and Epidemiology (IMBIE), University Hospital Bonn, 53127 Bonn, Germany; leonie.weinhold@ukbonn.de; 3 Division of Clinical Neuro-Oncology, Department of Neurology, University Hospital Bonn, 53127 Bonn, Germany; niklas.schaefer@ukbonn.de (N.S.); ulrich.herrlinger@ukbonn.de (U.H.); 4 Department of Anesthesiology and Intensive Care Medicine, University Hospital Bonn, 53127 Bonn, Germany; felix.lehmann@ukbonn.de (F.L.); christian.bode@ukbonn.de (C.B.); 5 Department of Geriatric Medicine and Neurology, Johanniter Hospital Bonn, 53113 Bonn, Germany; ahjacobs@uni-muenster.de

**Keywords:** meningioma, geriatric patients, prolonged mechanical ventilation, mortality

## Abstract

**Simple Summary:**

Meningiomas are most commonly benign intracranial tumors, and surgical resection represents the treatment modality of choice. However, especially for patients of higher age and with increasing comorbidities, brain surgery might be accompanied by the need for postoperative prolonged intensive care, which might impair intended operative benefit. In the present study, we therefore analyzed prolonged mechanical ventilation (PMV) as an indicator variable for such intensive care treatment with regard to potential effects on mortality after meningioma resection in patients aged 70 years and older. We found that patients with postoperative PMV exhibited a profoundly increased probability to die within 1 year after surgery. Based on these findings, we identified risk factors for postoperative PMV occurrence and developed an easy-to-use score which allows us to estimate the risk of PMV occurrence preliminary to the surgical resection. We believe that this score might help to more sufficiently guide patients in the course of risk–benefit assessment and preoperative counseling.

**Abstract:**

Indication for surgical treatment in patients with intracranial meningioma must include both clinical aspects and an individual risk–benefit stratification, especially in geriatric patients. Prolonged mechanical ventilation (PMV) has not been investigated for its potential effects in patients with meningioma. We therefore analyzed the impact of PMV on mortality in geriatric patients who had undergone meningioma resection. Between 2009 and 2019, 261 patients aged ≥ 70 years were surgically treated for intracranial meningioma at our institution. PMV was defined as postoperative invasive ventilation of >7 days. Postoperative PMV was present in 17 of 261 geriatric meningioma patients (7%). Twenty-five geriatric patients (10%) died within 1 year after surgery. A scoring system (“ACKT”) based on the variables of age, preoperative C-reactive protein (CRP) value, Karnofsky performance scale and tumor size supports prediction of postoperative PMV (sensitivity 73%, specificity 84%). PMV is significantly associated with increased mortality after surgical treatment of meningiomas in geriatric patients. Furthermore, we suggest a novel score (“ACKT”) to preoperatively estimate the risk of PMV occurrence, which might help to guide future risk–benefit assessment and patient counseling in the geriatric meningioma population.

## 1. Introduction

Within the aging population, an increasing percentage of intracranial meningiomas are diagnosed in the elderly. Microsurgical resection represents the treatment modality of choice for patients with intracranial meningioma. Nevertheless, aggressive surgical treatment of intracranial meningiomas remains controversial, especially in geriatric patients. In most cases, geriatric patients could benefit from surgery, but the risk of an unfavorable outcome is higher compared to the younger patient cohort [1]. This is partly due to additional pre-existing conditions, an increased likelihood of perioperative complications and the reduced physical resources of geriatric patients [2].

Therefore, the main goal of meningioma surgery for older people is to maintain life expectancy and quality of life while providing long-term alleviation/prevention of tumor-related complications. To achieve the best possible long-term outcome, adequate preoperative assessment, effective communication about the goals of the surgical intervention, reduction of the effects of surgical aggression and adequate perioperative management are essential. However, preoperative assessment of potential benefits of surgery for the elderly patient requires a detailed evaluation of individual chances of success. We have therefore evaluated our geriatric patient population with regard to possible risk factors for increased mortality after elective meningioma surgery. 

Patients with a postoperative prolonged mechanical ventilation (PMV) dependency have already been associated with high mortality rates in other etiologies [3,4]. Accordingly, we aimed to investigate the impact of PMV on mortality in geriatric patients with meningioma. Furthermore, we intended to develop a predictive score for the preoperative identification of a population of geriatric meningioma patients at risk of postoperative prolonged mechanical ventilation.

## 2. Results

### 2.1. Patient Characteristics

Between 2009 and 2019, 261 patients aged 70 years and older were surgically treated for intracranial meningioma at our department. Median age was 76 years (IQR 73–79), including 178 females (68%) and 83 males (32%; female/male ratio 2.1:1). Patients presented with a median preoperative Karnofsky performance scale (KPS) of 90 (IQR 80–90). Further details are given in Table 1. 

Preoperative symptoms at admission are given in Table 2. The most common preoperative symptom was tumor-associated epilepsy (23%), followed by cranial nerve deficits (15%) and sensory/motor deficit (11%). Twenty-three percent of the patients were asymptomatic.

### 2.2. Tumor Location/Extent of Resection/World Health Organization (WHO) Grade

The most frequent location of intracranial meningiomas in the present geriatric patient cohort was in the area of convexity (35%), followed by sphenoid wing (18%) and the falx (17%). There were 151 patients who presented with peritumoral edema (58%), and 25 patients harbored multiple meningiomas (10%, Table 1).

Regarding the extent of resection, 222 geriatric patients underwent Simpson grade I/II resection for meningioma (85%), while 39 patients underwent Simpson grade III/IV resection (15%). 

Tumor classification according to the WHO criteria included 187 geriatric patients with grade I (70%), 73 patients with grade II (28%) and 1 patient with grade III (0.4%, Table 1).

### 2.3. 1-Year Mortality

**Figure 1:**
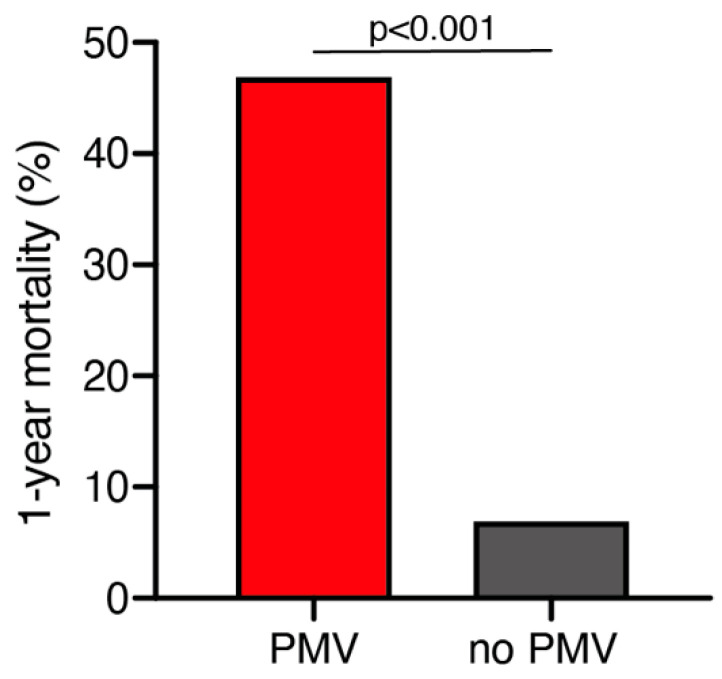
One-year mortality dependent on postoperative PMV occurrence. PMV, prolonged mechanical ventilation.

A total of 25 out of 261 geriatric patients (10%) died within 1 year after surgical treatment for intracranial meningioma. Patients who died within 1 year were significantly older compared to those still alive after 1 year (79 ± 6 years vs. 76 ± 4 years; *p* = 0.035, 95% CI 0.2–4.1). Patients who were still alive 1 year after the surgical treatment presented in a significantly better neurological condition according to preoperative KPS compared to patients who deceased within 1 year after surgical treatment (85 ± 14 vs. 76 ± 17; *p* = 0.005, 95% CI 2.6–14.7). Furthermore, patients who died within 1 year after initial surgical treatment were significantly more likely to suffer from postoperative PMV than those still alive during follow-up (47% vs. 7%; *p* < 0.0001, OR 11.9, 95% CI 4.1–34.7) (Figure 1). The median follow-up time was 23 months (IQR 7–42). Fourteen patients (5%) were lost to follow-up.

### 2.4. Postoperative Prolonged Mechanical Ventilation

In total, 17 of 261 geriatric patients (7%) suffered from PMV after surgical treatment for intracranial meningioma. Characteristics of patients with and without postoperative PMV after resection for meningioma are detailed in Table 3 and Supplementary Table S1. Patients with PMV presented with a significantly reduced initial mean KPS compared to patients without PMV (64 ± 18 vs. 85 ± 14; *p* < 0.0001, 95% CI 14.9–28.5). Patients with PMV harbored significantly larger tumors compared to patients without PMV (55 ± 24 mm vs. 38 ± 16 mm; *p* < 0.0001, 95% CI 9.3–25.5).

Preoperative corticosteroid prescription was significantly correlated with postoperative PMV (82% vs. 40%; *p* = 0.0007, OR 7.1, 95% CI 1.9–25.3). The mean preoperative C-reactive protein (CRP) value was significantly increased in patients with postoperative PMV compared to patients without PMV (23.9 ± 37.5 mg/L vs. 4.9 ± 12.3 mg/L; *p* < 0.0001, 95% CI 11.5–26.5). Preoperative CRP levels did not significantly correlate to the presence of preoperative corticosteroid medication: patients without corticosteroid intake exhibited a mean CRP value of 4.3 ± 12.5. Compared to this, patients with preoperative corticosteroid intake revealed a mean CRP value of 7.5 ± 18.04 (*p* = 0.8). The comorbidity index did not significantly differ between the groups of patients with and without PMV occurrence: 3 out of 21 patients with PMV (14%) compared to 14 out of 240 patients without PMV (6%) exhibited a CCI > 2 at admission (*p* = 0.1). Supplementary Table S2 provides an overview of comorbidity burden for the entire study population assessed by the CCI. 

Mortality after 1 year was significantly higher in patients with postoperative PMV compared to patients without PMV (47% vs. 7%; *p* < 0.0001, OR 11.9, 95% CI 4.1–34.7).

### 2.5. Predictive Score

**Figure 2:**
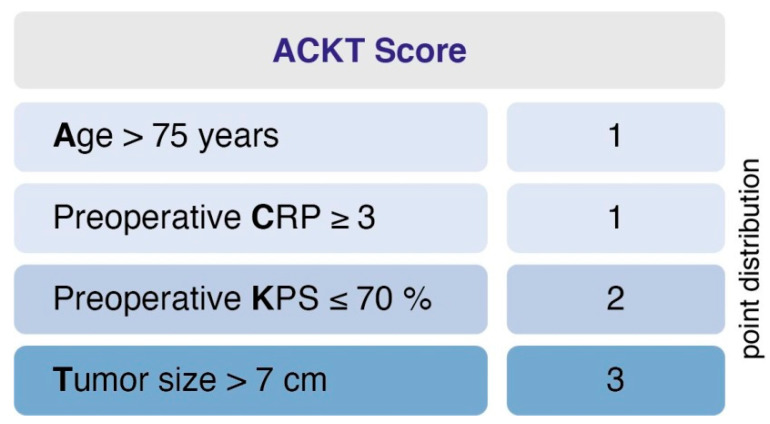
A clinical score to preoperatively estimate the risk of PMV occurrence in geriatric meningioma patients. An additive value of <3 implies a probability of 98% for not developing postoperative PMV. PMV, prolonged mechanical ventilation.

Further, we designed and evaluated a prediction score for PMV. The score was designed (1) to predict PMV based on easily identifiable and routinely recorded preoperative variables and (2) to be easy to calculate and implement. This yielded the following point distribution for a new score, which we called the “ACKT” score, ranging from 0 to 7 (Figure 2): age > 75 years (1 point); preoperative C-reactive protein (CRP) value ≥ 3 (1 point); preoperative KPS ≤ 70% (2 points); tumor size > 7 cm (3 points). In the present series, the mean score in patients with PMV was 3.7 (SD = 1.8) and 1.3 (SD = 1.2) in patients without PMV. Using a cut-off point of 3, the score yields a sensitivity of 73.3%, a specificity of 83.6%, a positive predictive value of 22.3% and a negative predictive value of 98%. An additive value of < 3 implies a probability of 98% for not developing postoperative PMV. Furthermore, the scoring system enables us to preoperatively demask 73% of patients that will suffer from PMV after intracranial meningioma resection.

## 3. Discussion

Intracranial meningiomas increase in their incidence with age [5,6]. Due to the higher life expectancy of the population, the management of benign brain tumors such as meningiomas thus becomes more important. In view of a potentially increased risk for the elderly at the time of resection of an intracranial meningioma, there is a growing demand for optimal preoperative patient selection in order to adapt clinical management at the earliest possible stage. Furthermore, in a typically elective setting, it is particularly important that patients and their relatives are provided with the most comprehensive consultation possible. Especially for elderly patients in good physical condition, the development of predictive scores in the past has led to an improvement in consultation possibilities. However, most of the research on this topic neglects potential complications associated with intensive care treatment. In the scope of other etiologies, the analysis of potential risk factors, which may lead to a prolonged mechanical ventilation period postoperatively, is already much more established compared to meningiomas [3,7,8,9]. 

### 3.1. Mortality in Geriatric Patients with Meningioma

The present study in patients aged 70 years and older demonstrated a 1-year mortality rate of 10% after meningioma resection. Although this number is within the range of the previous evaluations [10,11,12,13], it still represents a significant limitation that requires clarification and consideration. The purpose of an elective surgical intervention in advanced age should be the preservation of quality and duration of life [14]. Therefore, various predictive models and data analyses have been attempted in recent years to better estimate the morbidity and mortality of geriatric patients with intracranial meningiomas [10,13,15,16,17]. Complementing these conclusions, the present study detected for the first time postoperative PMV as a significant factor associated with increased 1-year mortality. Since long-term mechanical ventilation is likely to be associated with extended hospitalization and less favorable re-convalescence of the patient, it is quite reasonable that in the multivariate analysis with regard to 1-year mortality, PMV was identified as a significant and independent predictor. However, the negative influence of PMV on survival, especially in a geriatric patient population, is most certainly not exclusively attributable to the potentially adverse effects of intensive care treatment. Especially in the subgroup of geriatric patients, there is a highly differentiated confrontation with potential future necessity for intensive care, which in geriatric patients with PMV might result in an implementation of palliative care or withdrawal of mechanical ventilation in order to respect the patients’ wishes [18].

In the present series, the proportion of 58% of meningioma patients with additional peritumoral edema is at the upper range of reported data on brain tumor edema occurrence [19,20,21]. However, it is important to mention that in the present series, only patients who had undergone surgical resection of the meningothelial tumor lesion were included for further analysis. Given the fact that profound peritumoral edema often contributes to impaired neurological and functional status, and therefore may lead to the indication for meningioma resection, the incidence of brain tumor edema in patients that undergo meningioma surgery may supersede respective rates in the entirety of patients with intracranial meningioma.

### 3.2. Prolonged Mechanical Ventilation in Geriatric Meningioma Patients

The need for prolonged mechanical ventilation has been reported as a determinant of a morbid postoperative course, especially in patients who have undergone cardiac surgery [3,4]. Malek and co-authors recently noted that a conglomerate of mainly preoperative patient-specific factors may account for a “larger-than-anticipated” involvement in PMV [22]. Therefore, more attention should be devoted to the preoperative identification and management optimization of high-risk patients. In contrast to patients with a severe cardiac condition, patients with intracranial meningiomas often allow elective planning of surgery, except in the case of corresponding neurological deficits [23]. Data on the influence of PMV and its risk factors have not yet been thoroughly addressed within the neurosurgical patient population. In the present study on geriatric patients with intracranial meningiomas, 7% of patients suffered from the need for postoperative PMV. Thus, the occurrence of PMV was associated with an impaired preoperative functional constitution, an increased CRP value and a larger tumor size. Furthermore, preoperative corticosteroid medication was also independently associated with an increased incidence of PMV. Though the use itself, the appropriate time point of beginning, as well as the appropriate dosage are still controversially debated, preoperative administration of corticosteroids is mostly applied to reduce tumor-related perifocal brain edema and has also been demonstrated to improve cardiopulmonary compliance and oxygenation in patients with acute respiratory distress syndrome (ARDS) [24,25,26]. Therefore, the demonstrated association seems to be paradoxical. There is a debate as to whether preoperative corticosteroid administration exerts an immunoregulatory effect, especially within a geriatric cohort [27]. However, the possible involvement of a confounding bias remains evident, since preoperative administration of corticosteroids in most meningioma patients is only prescribed in the presence of certain clinical/imaging findings. 

### 3.3. ACKT—A Predictive Score for PMV in Geriatric Meningioma Patients

This study provides a novel scoring system to predict prolonged mechanical ventilation after meningioma surgery in geriatric patients. Our risk index includes the use of four easily identifiable and routinely recorded preoperative variables to predict prolonged mechanical ventilation after meningioma resection in geriatric patients.

The results presented in this study have several implications. They could be relevant for clinical management, as well as for future research on respiratory complications in neurosurgical patients. Furthermore, they may lead to various changes in the perioperative management of geriatric meningioma patients and may allow for better preoperative discussion/consultation and consensus building on the assumed risk/benefit ratio of the proposed surgical approach versus observational practice.

## 4. Materials and Methods

### 4.1. Patients

Between 2009 and 2019, 261 patients aged ≥ 70 years were surgically treated for intracranial meningioma at our institution. Review of records was performed retrospectively after institutional review board approval had been obtained. Pertinent clinical information including age, sex, KPS, presence of comorbidities, preoperative laboratory values, tumor localization, tumor size and presence of peritumoral edema, American Society of Anesthesiologists (ASA) classification, WHO grade referring to postoperative histological examination, extent of tumor resection according to the Simpson grading system and postoperative complications were collected and entered into a computerized database (SPSS, version 25, IBM Corp., Armonk, NY). To determine tumor size, a diameter-based approach was used in which the single largest diameter on a single axial preoperative contrast-enhanced T1 MR slice was selected [28]. Peritumoral edema was defined as a difference of more than 1 cm between the maximum diameter of the meningioma surrounded by the edema and the meningioma itself in FLAIR-weighted MRI sequences [23,29]. Preoperative C-reactive protein (CRP) levels were stratified into values ≥ 3 mg/L and < 3 mg/L, as this cut-off marks the discrimination between physiologic and pathologic CRP levels according to the diagnostic laboratory at the University Hospital Bonn. Decision on preoperative steroid intake as well as duration of corticosteroid use was mainly based on the opinion of the pretreating colleagues in view of individual treatment regimes as well as patient-specific characteristics and did not adhere to a uniform standard. Comorbidity burden was determined on the basis of the Charlson Comorbidity Index (CCI) and dichotomized into patients with a low (CCI < 2) and a high comorbidity burden (CCI > 2) as previously described [2,30]. Indication for brain tumor resection was based on interdisciplinary decision making in the course of weekly CNS tumor board meetings held by specialists in the field of neurology, neurosurgery, neuroradiology, neuropathology and radiation oncology, as described previously [2]. Thereby, decision for brain tumor surgery was made for diagnostic reasons, to prevent new onset or worsening of existing focal neurological deficits as well as to prevent malignant obstructive hydrocephalus and/or progressive compression or impairment of adjacent physiological brain areas by the meningioma lesion and the tumor-related brain edema. 

KPS was used to evaluate patients during admission and 3 months postoperatively, according to their functional status. At admission and 3 months after surgery, patients with intracranial meningioma were divided into KPS ≥ 70% vs. < 70%. With regard to the ASA classification, a further stratification was carried out, dividing the patients into a group with ASA 1 or 2 and a group with ASA 3 or 4. Postoperative complications were defined as postoperative events that required additional surgical treatment within 30 days after initial meningioma resection, as well as postoperative events that had to undergo conservative therapy [31].

PMV was defined as an invasive ventilation period of >7 days after meningioma resection [3].

Histopathological grading was performed according to the 2016 WHO criteria [32]. All previous pathology reports underwent renewed review to confirm that diagnosis was in keeping with these requirements. Patients underwent standardized preoperative clinical, computed tomography (CT) and magnetic resonance imaging (MRI) examinations. Clinical and imaging follow-up consisted of MRI scans 3 months after surgery, as well as a yearly imaging for the following 5 years. Earlier clinical and imaging evaluation was advised in case of new or worsened neurological deficits as well as radiological signs of tumor recurrence or progression. 

The primary endpoint of our study was to examine risk factors for 1-year mortality. Another goal was to examine risk factors for PMV, as well as to develop a prediction model. The primary endpoint of our study was to unveil a potential correlation between postoperative PMV and 1-year mortality. In a second step, we aimed at developing a predictive scoring system which may enable us to preoperatively assess the risk of PMV occurrence in geriatric patients who undergo brain surgery for intracranial meningioma.

### 4.2. Predictive Scoring Model

With regard to the 17 patients with postoperative PMV, variables that might differ in reality between the two groups of patients with postoperative PMV occurrence and without may not find a statistically significant difference based on the available data. Therefore, a mere inclusion of variables in a predictive score which reaches statistical significance in a univariate analysis may lose possibly valuable information regarding the prediction of PMV and was avoided in accordance with Heinze et al. [33]. This led to the establishment of a scoring based on clinical expertise.

### 4.3. Statistical Analysis

In addition to the above-described predictive model analyses, data analyses were performed using the computer software package SPSS (version 25, IBM Corp., Armonk, NY, USA) and R version 4.0.3 [34]. Mann–Whitney U-tests were used to compare continuous variables. Categorical variables were analyzed in contingency tables using Fisher’s exact test. Results with *p* < 0.05 were considered statistically significant. To find potentially independent predictors for 1-year mortality and PMV in geriatric patients undergoing surgical treatment for intracranial meningioma, logistic regression models were performed. All variables with significant *p*-values in the univariate analysis were considered as potentially independent variables in the backward elimination process. 

## 5. Limitations

The retrospective design with data from only one center reduces the generalizability of our prediction score. Without external validation, the proposed scoring model does not exhibit enough power to enable strong risk stratifying in geriatric meningioma patients. It is therefore important not to mistake the authors‘ intended attempt of a first-time proposal for a potential future risk–benefit assessment as a proposal for a new treatment guidance for geriatric meningioma patients. Further external and prospective evaluation is needed to sufficiently validate the score and the cut-off point. Possible collinearities of different factors cannot be excluded due to the strong patient selection and focus only on geriatric patients. Despite these deficiencies of the available data and their interpretation, to the best of our knowledge, this is the first attempt of this kind in geriatric patients with surgical treatment of intracranial meningiomas. Further research and external, prospective validation of our prediction model is highly necessary.

## 6. Conclusions

The present study demonstrates a strong correlation of postoperative PMV and increased mortality in geriatric meningioma patients. We further suggest a novel score (“ACKT”) which might provide a guide for preoperative assessment of the risk of PMV development in the course of meningioma surgery in the geriatric patient cohort, and thus might improve preoperative patient counseling and risk–benefit assessment.

## Figures and Tables

**Table 1:** Patient characteristics.

No. of Patients	261
**Median age (IQR) (in y)**	76 (73–79)
**Sex**	
Female	178 (68)
Male	83 (32)
**Median preoperative KPS (IQR)**	90 (80–90)
**Tumor location**	
Convexity	91 (35)
Sphenoid wing	47 (18)
Falx	44 (17)
Frontobasal	27 (10)
Posterior fossa	13 (5)
Others	39 (15)
**Multiple meningioma**	25 (10)
**Peritumoral edema**	151 (58)
**Simpson grade**	
Simpson grade I/II	222 (85)
Simpson grade III/IV	39 (15)
**WHO grade**	
WHO grade I	187 (70)
WHO grade II	73 (28)
WHO grade III	1 (<1)

* Values represent number of patients unless otherwise indicated (%). IQR, interquartile range; KPS, Karnofsky performance scale; SD, standard deviation; WHO, World Health Organization; y, years.

**Table 2:** Preoperative symptoms. Values represent number of patients unless otherwise indicated (%).

Symptoms	No. of Patients
**Asymptomatic**	**59 (23)**
**Seizures**	**59 (23)**
**Cranial nerve deficit**	**39 (15)**
**Sensory/motor deficit**	**30 (11)**
Sensory deficit	2 (7)
Motor deficit	25 (83)
Combined	3 (10)
**Cephalgia**	**22 (8)**
**Gait disorder**	**21 (8)**
**Vertigo**	**17 (7)**
**Aphasia**	**14 (5)**

**Table 3:** Factors associated with PMV following intracranial meningioma resection.

	Pts. w/o Postoperative PMV	Pts. with Postoperative PMV	*p*-Value
**No. of patients**	244 (93)	17 (7)	
**Female**	168 (69)	10 (59)	0.42
**Median age**	76 (73–79)	78 (76–81)	0.07
**Median preoperative KPS**	90 (80–90)	70 (50–80)	<0.0001
**Tumor location**			0.48
Convexity	84 (34)	7 (41)	
Sphenoid wing	44 (18)	3 (18)	
Falx	43 (18)	1 (6)	
Posterior fossa	13 (5)	0 (0)	
**Multiple meningioma**	23 (9)	2 (12)	0.67
**Tumor size (in mm)**	38 ± 16	55 ± 24	<0.0001
**Preoperative anticoagulant medication**	78 (54)	3 (18)	0.3
**Preoperative corticosteroid medication**	97 (40)	14 (82)	0.0007
**Mean preoperative CRP (in mg/L) (+/-SD)**	4.9 ± 12.3	23.9 ± 37.5	<0.0001
**Extent of resection**			0.15
Simpson grade I and II	210 (86)	12 (71)	
Simpson grade III and IV	34 (14)	5 (29)	
**WHO grade**			0.4
WHO grade I	177 (73)	10 (62)	
WHO grade II and III	68 (27)	6 (38)	
**1-year mortality**	17 (7)	8 (47)	<0.0001

* Values represent number of patients unless otherwise indicated (%). No., number; Pts., patients; w/o, without; PMV, prolonged mechanical ventilation; SD, standard deviation; y, years; KPS, Karnofsky performance scale; WHO, World Health Organization.

## Data Availability

Data is contained within the article or supplementary material.

## Supplementary Materials

The following are available online at https://www.mdpi.com/2072-6694/13/1/98/s1, Table S1: Characteristics of patients with PMV and meningioma. Table S2: Frequency of Charlson Comorbidity Index conditions (*n* = 261).
