# Supplementary material for: ACKT: A Proposal for a Novel Score to Predict Prolonged Mechanical Ventilation after Surgical Treatment of Meningioma in Geriatric Patients
Source: Cancers (Basel). 2020 Dec 31;13(1):98. doi: 10.3390/cancers13010098 (PMC7795978; doi:10.3390/cancers13010098)
Supplement: Supplementary file 1 [file cancers-13-00098-s001.pdf]

cancers

Supplementary Materials: ACKT: A proposal for a novel score to predict prolonged mechanical ventilation after surgical treatment of meningioma in geriatric patients

Elisa Scharnböck, Leonie Weinhold, Anna-Laura Potthoff, Niklas Schäfer, Muriel Heimann, Felix Lehmann, Erdem Güresir, Christian Bode, Andreas H. Jacobs, Hartmut Vatter, Ulrich Herrlinger, Matthias Schneider and Patrick Schuss

Table S1. Characteristics of patients with PMV and meningioma.

Patient No.	Age, Sex	Duration of PMV (h)	CCI	ASA	Postoperative complication	Postoperative Status epilepticus
1	76, f	467	2	2	hemorrhage	no
2	77, m	525	1	2	brain swelling	no
3	78, f	841	1	2	seizures	yes
4	79, f	538	1	2	hemorrhage	no
5	82, m	1557	1	2	SVT, SSI	no
6	89, m	340	2	2	hemorrhage, PE	no
7	72, f	420	1	3	pneumonia	no
8	73, m	582	1	3	pneumonia	no
9	76, f	240	1	3	brain swelling, PE	no
10	78, m	199	1	3	SSI, pneumonia	no
11	78, f	169	1	3	SSI, PE	no
12	79, f	409	1	3	seizures	yes
13	80, f	527	1	3	hemorrhage	no
14	85, m	338	1	3	pneumonia	no
15	94, m	171	1	3	pneumonia	no
16	70, f	454	1	4	brain swelling, PE	no
17	78, f	339	2	4	seizures	yes

PMV, prolonged mechanical ventilation; CCI, Charlson comorbidity index; ASA, American Society of Anesthesiologists; SVT, sinus vein thrombosis; SSI, surgical site infection; PE, pulmonary embolism.

Table S2. Frequency of Charlson Comorbidity Index conditions (n = 261).

Index weight	Condition	Frequency % (n)
1	Coronary artery disease	3 (8)
1	Congestive heart failure	2 (6)
1	Peripheral vascular disease	1 (3)
1	Cerebrovascular disease	3 (8)
1	Dementia	0 (0)
1	Chronic pulmonary disease	9 (23)
1	Connective tissue disease	0 (0)
1	Ulcer disease	0 (0)
1	Mild liver disease	0 (0)
1	Diabetes	20 (52)
2	Hemiplegia	8 (21)
2	Renal disease	2 (5)
2	Diabetes with endorgan damage	0 (0)

2	Any tumor	3 (9)
2	Leukemia	0 (0)
2	Lymphoma	0 (0)
3	Moderate/severe liver disease	0 (0)
6	Metastatic solid tumor	1(2)
6	AIDS	0 (0)
